# Sex Disparity Among Canadian Cardiologists in Academic Medicine: Differences in Scholarly Productivity and Academic Rank

**DOI:** 10.7759/cureus.18687

**Published:** 2021-10-11

**Authors:** Jacqueline Rano, Sabeena Jalal, Tara Sedlak, Javed Butler, Muhammad S Khan, Warren J Manning, Faisal Khosa

**Affiliations:** 1 Medicine, Royal College of Surgeons in Ireland - Medical University of Bahrain (RCSI - MUB), Busaiteen, BHR; 2 Medical Education and Simulation, Vancouver General Hospital, Vancouver, CAN; 3 Radiology, Vancouver General Hospital/University of British Columbia, Vancouver, CAN; 4 Medicine, University of British Columbia, Vancouver, CAN; 5 Medicine, University of Mississippi Medical Center, Jackson, USA; 6 Internal Medicine, John H. Stroger, Jr. Hospital of Cook County, Chicago, USA; 7 Medicine (Cardiovascular Division), Beth Israel Deaconess Medical Center, Harvard Medical School, Boston, USA; 8 Radiology, Beth Israel Deaconess Medical Center, Harvard Medical School, Boston, USA

**Keywords:** h-index, research publications, academic cardiology, academic rank, sex disparity

## Abstract

Background

Women remain relatively underrepresented in all subspecialties of academic medicine. While sex disparity is prevalent in a number of specialties, the association between academic productivity and sex in academic cardiology has not been assessed in the Canadian context.

Methods

Academic faculty of accredited Canadian Resident Matching Service (CaRMS) programs were included from cardiology division websites across 17 universities. Cardiology faculty members’ names, academic ranks, leadership positions, and sex were obtained from each institutions’ website. The Elsevier database Scopus© was used to extract the Hirsch index (H-index), years of active research, and number of publications of each faculty member. The H-index was used as a metric of academic output and research productivity. Univariate regression was run with the H-Index as the outcome of interest, and multiple linear regression analysis was used to determine factors associated with higher H-index.

Results

Sex was identified for 1,040 members, of whom 836 (80%) were male. Male members had higher numbers of publications (p <0.001). There was a trend for males in a leadership position to have a higher H-index (p = 0.07). Median H-index was lower for women (p = 0.02). Males across assistant and associate professor ranks had a higher H-index. Women achieving professor rank demonstrated greater productivity with a higher median H-index (p = 0.002).

Conclusions

There is a prevalent sex gap in academic cardiology with regard to scholarly productivity and academic achievement. Factors that may help narrow the sex gap need to be identified and corrective measures implemented to enhance sex equity.

## Introduction

Women are underrepresented in academic medicine [1-3]. Women in academic medicine are less likely to participate in research, have lower salaries [4], and report less professional satisfaction in academic practice [1]. Recent publications have also documented that women are underrepresented in senior academic ranks and leadership positions in academic disciplines [5,6], professional societies [7], and editorial boards of medical journals [8]. Fewer women advance to the professor rank [9], and do so at a slower rate [10]. Female acceptance rates to United States medical schools have increased from 8.9% in 1965 to 47.9% in 2016 [9]. However, the percent of women entering cardiology programs continues to be substantially lower than the percentage of women entering medical schools [4].

Women are underrepresented in cardiology in the United States [4,11] Canada [12], the United Kingdom [13], and Europe [14]. As of 2019, males comprise 78% of the cardiology workforce in Canada [12]. More male than female cardiologists practice academically. Women are further underrepresented in academic leadership positions including chair, director, or division program head. This disparity regarding leadership is evident within cardiology in the United States, where the number of males holding an academic cardiology position as program director or division chief in 2018 greatly outnumbered females [15]. Female cardiologists have lower salaries [16] and report less satisfaction with financial compensation, especially in academic positions [17]. Although reports have shown high career satisfaction in both male and female cardiologists [18], women in academic medicine have been more dissatisfied with their ability to achieve their professional goals [19].

The objective of this study was to identify sex differences in academic rank, productivity, and leadership among cardiology faculty within Canadian cardiology programs. With knowledge of current sex inequities, program policies and procedures can be developed to enhance sex equity.

## Materials and methods

A cross-sectional analysis study was conducted discerning sex and research productivity among academically affiliated cardiologists in Canada. Informed consent or institutional review board approval was not requested as data were collected from publicly available sources. Data were collected from university department websites and Elsevier’s® Scopus© database during April of 2018. Academic faculty members of cardiology and cardiac science departments of all 17 accredited Canadian Resident Matching Service (CaRMS) programs were included. Department websites were accessed for each program and faculty listings retrieved (Appendix). Data pertaining to academic ranks, sex, and leadership positions were recorded from each institution’s website. Sex was assessed using department name and faculty photo and cross-checked with each respective province’s college of physicians and surgeon’s public physician directories. There were no discrepancies found. Sex demographics were not available on the directory for two provinces, Manitoba and Newfoundland and Labrador, and sex was retained using name and faculty photo. Leadership ranks (e.g., division head or director, such as ‘director of electrophysiology’) were recorded. No distinction was made to leadership rank and all were recorded as holding a leadership position. This methodology has been used in several recent publications [20,21]; however, it has not been validated against a gold standard.

The inclusion criteria for this study included faculty who held a Doctor of Medicine degree in Canada (or equivalent) and were a cardiologist who held an academic rank within the university. Academic ranks were recorded from lowest to highest order of faculty tenure track beginning assistant professor, associate professor, and full professor. Individuals were coded based on their primary area of practice being in adult or pediatric cardiology, as listed. Administrative staff members were excluded. Adjunct professors, emeritus professors, residents, fellows, cardiac radiologists, and cardiac anesthesiologists were excluded. In some cases, variations in academic rank were found, and the highest rank was used for the analysis.

The biomedical database Scopus was used to gather data regarding an individual’s Hirsch index (H-index), number of citations, number of publications, and years of active research. Scopus is a reliable and up-to-date database maintained by Elsevier to assess these variables [22]. The H-index, a single numerical metric provided by the Scopus database, has been shown to be a reliable measure of an author’s academic research productivity and citation impact. The H-index takes items into account, such as number of publications and citations [23]. The H-index has been positively correlated with academic rank across multiple specialties [2,6,21]. H-index was used in this study as a method of quantitating academic output and impact. Years of active research provides an estimate of the time period in which an author has been engaged in research. Years of active research was calculated as the difference between the first and the most recent publication dates.

Sex was our primary exposure of interest. Each variable was regressed independently with H-index as the outcome of interest. Assumptions were checked and significance was reported. Median and ranges were noted for quantitative variables (non-transformed variables). Frequency and percentages were noted for the qualitative variables. Multicollinearity between independent variables was assessed using a correlation coefficient. Cramer’s V test was used for one nominal and one ordinal variable, and Spearman’s test was used for one continuous variable and one ordinal variable. A correlation of 0.9 was treated as the presence of multicollinearity. Histograms and the Kolmogorov-Smirnov test were used to assess for normal distribution. The Wilcoxon rank-sum test and Kruskal-Wallis test were used to test whether there was a difference in the groups or not. Linear regression was run to study the effect of covariates on the H-index. At univariate level, simple linear regression was applied using F-test statistics. Based on results of the univariate regression, variables were taken forward in the multivariable analysis based on the cut-off value of 0.25. Main effects were identified using a stepwise selection strategy, which was used to build the model, and based on the p-value, variables with significance (p≤0.05) were included. Partial F-test was used to compare the models. The final step was to check for interaction (cut-off value of 0.1). Interaction terms were created between each of the main effects in the model, including citations, academic rank, years of research, and leadership. Interaction was checked by creating pair-wise interactions between independent variables and checking them by putting them in the model one by one and looking at their p-value and comparing it to the level of significance. The level of significance for checking the interaction was at alpha = 0.10. Finally, logistic regression was run to calculate the odds ratio (OR). Sex was taken to be the dependent variable when logistic regression was run, and OR was reported. Data were analyzed using STATA (version 14.2, StataCorp LP, College Station, Texas).

## Results

Academic cardiologists were identified and recorded in 17 Canadian universities from eight provinces (Table 1). These included 1,040 cardiologists for whom information about sex was available, of which 836 (80.4%) were males. In academic cardiology, there were a significantly greater number and a higher proportion of practitioners who were male (p < 0.001). All universities had a majority male representation (Table 1). There were 923 (88.8%) adult cardiologists, of which 758 (82.1%) were males. There were 117 (11.3%) pediatric cardiologists, of which 78 (66.7%) were males. Academic ranks were identified for 910 (88%) cardiologists. Among all ranks, the proportion of males was significantly higher (p = 0.001). When sex was stratified by academic rank, females comprised the least proportion of full professors for this data set (13.5%), followed by associate professors (17.4%), with the greatest representation in the assistant professor strata (26.3%) (Table 1).

**Table 1:** List of Canadian universities, provinces, specialties and academic ranks included with % mode for each (total n, % male) SK, Saskatchewan; MB, Manitoba; NFL, Newfoundland; NS, Nova Scotia; BC, British Columbia; AB, Alberta; ON, Ontario; QC, Quebec

	n	%
University		
University of Saskatchewan	19	84
University of Manitoba	29	79
Memorial University Newfoundland	25	88
Dalhousie University	58	79
University of British Columbia	67	81
University of Alberta	88	80
University of Calgary	91	78
McMaster University	56	84
University of Toronto	203	80
University of Ottawa	45	76
Université de Sherbrooke	38	79
Western University	38	92
Queens University	21	86
Université Laval	50	84
McGill University	45	73
Université de Montréal	148	79
Northern Ontario School of Medicine	20	80
Province		
SK	19	84
MB	29	79
NFL	25	88
NS	58	79
BC	67	81
AB	179	79
ON	383	82
QC	281	79
Primary area		
General adult cardiology	923	82
Pediatric cardiology	117	67
Academic rank		
Assistant professor	403	74
Associate professor	270	83
Full professor	237	87

**Figure 1:**
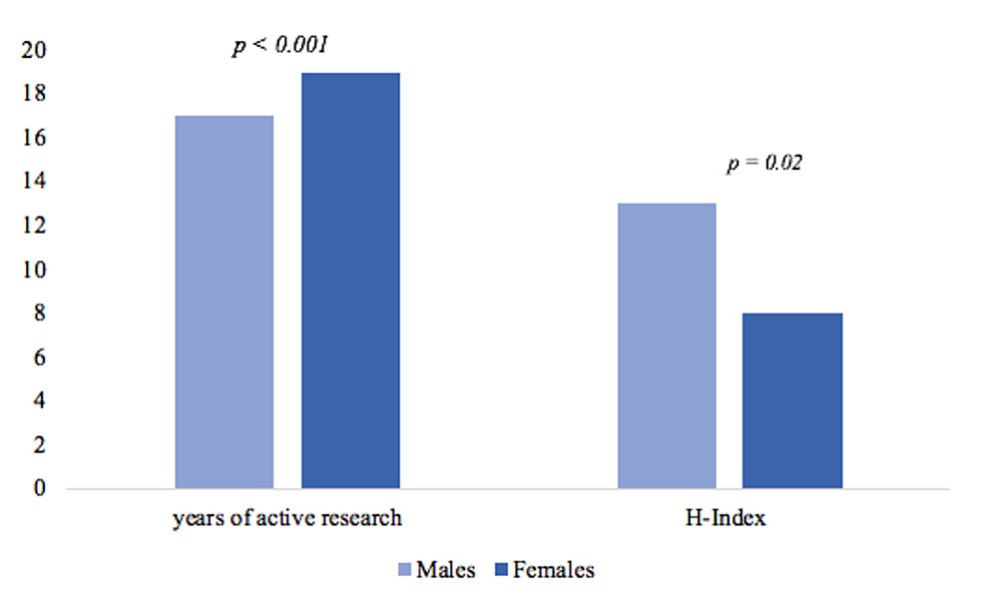
Sex analysis for median H-index and years of active research

**Figure 2:**
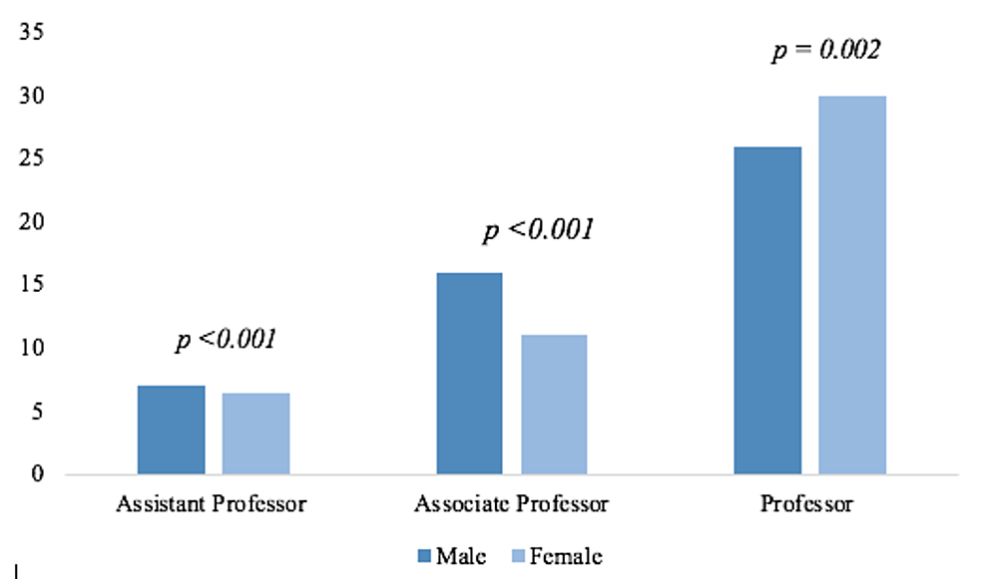
Median H-index of males and females across academic rank

Median H-index differed between sexes. Median H-index was lower for female cardiologists overall (p = 0.02); median H-index was 13 for males and 8 for females. Median H-index was higher for males (12.5) than females (7) across pediatric cardiology (p = 0.04) but not adult cardiology (p = 0.11), where the median H-index was 12 for males and 9 for females. On average, male cardiologists had a higher number of publications (p < 0.001), with a median of 32 publications, whereas female members had 19.2 publications. Median years of active research was higher for females (p <0.001) (Figure 1), where males had a median of 17 years and women had a median of 19 years of active research. Years of active research did not have a strong correlation with H-index (r = 0.16). Median H-index of academic ranks was greater for males across the assistant professor rank (p ≤ 0.001), where males had a median H-index of 7 and women had a median H-index of 6.5. Median H-index was also greater for males across the associate professor rank (p ≤ 0.001), where males had a median H-index of 16 and women had a median H-index of 11. However, female professors had a significantly higher (p = 0.002) median H-index (30) than males (26) (Figure 2). There were 367 (35.3%) individuals in leadership roles, of whom 295 (80.4%) were males (p < 0.001). There was a trend for males in a leadership position to have a higher median H-index (p = 0.07), where the median H-index of males in a leadership position was 18 compared to women in a leadership position who had a median H-index of 13.

Univariate regression was run with H-index as the outcome of interest. Significance was found for sex (p = 0.02), university (p = 0.04), and academic rank (p = 0.02). We had checked for collinearity, with a cut-off of 0.08. We found collinearity between the variables citations and publications and we removed the variable citations from the multivariable model. Based on results of the univariate regression, the variables that were taken forward in the multivariable analysis were as follows: sex (p = 0.542); university (p = 0.021); publications (p ≤ 0.001); years of research (p = 0.04); academic rank (p = 0.043); and leadership (p = 0.049) (Table 2). There were no significant interactions. The prediction equation used accounted for major variability in our final model as follows. Female members had lower odds of having a higher H-index if we adjusted for citations, academic rank, years of research, university, and leadership, which was demonstrated by an adjusted R square = 0.81 and p ≤ 0.001. This shows that 81% of the variability in this model is explained by this model. Logistic regression was run to calculate the OR. Sex was taken to be the dependent variable. The odds of females having a lower H-index than males was 0.68.

**Table 2:** Multivariate regression

	Coefficient	Standard error	p-Value	Lower limit 95% CI	Upper limit 95% CI
Sex: males (ref: female)	-1.830	2.99	0.542	-7.717	4.056
Academic rank					
Assistant professor	-1.614	5.978	0.787	-13.34	10.119
Associate professor	-0.985	6.300	0.52	-13.351	11.38
Full professor	1.58	6.089	0.043	1.55	10.391
Publications	0.129	0.0238	<0.001	0.0820	0.1757
Years of active research	0.0003	0.0003	0.04	0.0002	0.0009
University	0.587	0.378	0.021	0.154	1.328
Leadership	1.728	2.551	0.049	1.279	6.734

## Discussion

In this study of Canadian academic cardiology faculty, we found that women are underrepresented in numbers among academic ranks and were found to exhibit less academic research productivity, as demonstrated by lower H-indices as well as fewer publications. Similar findings of sex disparity have been reported in Canada among academic leadership positions in radiology [20,24], physical medicine [21], and dermatology [3]. We observed the lowest proportions of women at the full professor rank, with the highest proportion of women at the assistant professor rank. Are qualified women experiencing systemic gender inequity as a result of a biased academic promotion process? Interestingly, the median H-index (academic research productivity) of academic ranks was significantly higher for women who achieved full professor status. This brings forth the discussion of conceivable explanations for why female cardiology professors demonstrate more research productivity than their male counterparts.

Female professors who have taken family leave during their career may academically mature later than males. Similarly, those who take family leave may have retained motivation and enthusiasm for a longer period. In contrast, males may have depleted efforts earlier after decades of uninterrupted work. Alternatively, women who obtain such positions may be more qualified or require more evident scientific productivity prior to hiring than their male counterparts. The scholarly duties of academic clinicians are also broad and include both teaching and research components. Lower H-index by academic rank may be explained by differences in time dedicated to research versus teaching. Many individuals, and perhaps more women, have teaching as a greater portion of their academic focus. Women may engage less in research during the tenure ranks of assistant and associate professor. The later period of one’s career, where family responsibilities decline, might allow for one to re-engage or partake with more involvement in research productivity. This may coincide with one reaching the rank of full professor.

Alternatively, our results may be indicative of the successful implementation of affirmative action policies in recent years. The finding of female professors demonstrating higher H-index may be an early sign of a trend already in place from proactive department initiatives. In 1995, a highly cited study reported that females in academic medicine had published less, and only 5% of women (compared to 23% of men) reached professor, while rank differences were not justified by academic productivity differences [25]. Even though our results show that males had higher numbers of publications, women in professor rank were found to be more productive. More recent research in 2016 has presented that women in academic medicine who do advance in academic rank, do so at a slower rate [10]. In our study, we observed that investing more years in active research did not have a strong correlation with H-index. Cardiologists who demonstrated greater productivity produced more research in fewer years. Also, even though the median years of active research was higher for females, the median H-index was lower for females (Figure 1). We must, however, acknowledge that one’s years of active research fails to account for the interruptions in academic activities resulting from parental leave. Although Canada has fairly inclusive parental leave policies that allow both parents to divide time off work, it remains the societal norm that a mother takes the majority of the parental leave time (in opposite-sex relationships). For an average family with two children, this would account for up to two years with decreased or no research productivity, not accounting for the time needed to 'catch up' upon return to work. This may account for an undeterminable portion of the differences between the H-indices between men and women.

Over a third of all cardiologists held a leadership role, with the threshold less than 20% for women. However, the distribution of leadership roles was observed to be appropriately represented by gender. The proportion of 80.4% of all leadership positions being held by males was found to be equivalent to the total gender distribution of 80.4% males. Therefore, with the current proportions of women, it is not realistic to expect women to hold 50% of leadership titles or to conclude that women are not obtaining sought-after roles in academic cardiology. There was an observed trend for women in a leadership role to have a lower H-index (p = 0.07), demonstrating less productivity. Similarly, male program directors in academic cardiology in 2018 were found to have a higher number of publications, have a higher H-index, and be of higher academic rank [15]. Our observed result in leadership differences may be due to family responsibilities, which traditionally have been deemed a female barrier to academic advancement.

Regarding lifestyle choices and family responsibilities, differences have been well documented. In 1996 [19] and more contemporaneously [16], women are found to be more likely to work part-time. Also, women in cardiology have reported family responsibility as negatively affecting their career [11]. Contributing factors to promotional success have been identified. Lack of efficient mentoring has been consistently identified as a limitation for female cardiologists [13,21]. Alternatively, sex differences in a work setting may impact one’s academic footprint/output. For instance, women may be more likely to work in community practice while still holding academic affiliation. In 2017, 45% of Canadian cardiologists reported their primary work setting as an academic health sciences center [12], therefore nearly half of the workforce practices within an academic setting. Unfortunately, we have no data on the sex breakdown of work setting.

Aside from the suspect of systematic barriers and differences in lifestyle choices, female cardiologists in Canada have reported discouragement and lack of encouragement [26]. Female cardiologists in the United States reported more sex-related workplace discrimination [18], 11. In one study, 30% of female medical faculty members reported having experienced sexual harassment, with 47% indicating their career advancement was negatively affected as a result [27]. In an era where modern movements have brought forth openness, reporting experiences of the present day are as complex in medicine just as in any other field, where academic medicine falls under the #MeToo umbrella [27,28]. As such, existing sex disparity may be independent of eagerness to climb the promotional ladder. An important consideration is women who do go on to reach full professor rank may considerably differ in experiences of professional barriers (such as harassment) from those who did not, and this may represent a biased sample in itself.

It should also be considered that our findings may be attributed to personal preference in time dedicated to scholarly endeavors. Full professorship and leadership titles are not universal goals. Both the true trajectory and desired trajectory of an individual’s academic career path are difficult to characterize. Further research should identify women who advance to professor in cardiology and assess which factors were attributed to their perceived success. More specifically, it is important to capture the nature of academic progression as a time-based evolution. This would yield a comparison of the slope rise of research productivity and achievement versus time for males versus females. Academic maturation through academic rank, administrative responsibility, presentations, and publications over time is not currently known. A longitudinal study of the progression of male versus female careers is therefore needed.

Suggestions have been made to improve the disparity found herein. Guidelines should accommodate work-life balance for both female and male residents and faculty members, as episodes of training discontinuity for pregnancy or child-rearing may delay academic maturity. A return-to-work parent model for mothers who have children during emergency medicine residency was recently implemented as official policy following a successful pilot study in 2018 at Stanford University [29]. The policy included provisions such as provisionally eliminating overnight shifts with subsequent removal of mothers’ names otherwise included in a sick call coverage pool in consideration of difficulties organizing short notice childcare [29]. A policy similar to this may be beneficial for Canadian cardiology residents to balance work and professional life. Women should be encouraged to pursue the diverse array of cardiac specializations, and appropriate mentorship should be systematically implemented at a local and personal level.

Limitations

Our study has several limitations. Elsevier’s Scopus database was used to extract data regarding scholarly output. The assumption that cardiologists not present on Scopus have no published academic work may be erroneous. Credibility for work can be lost due to name changes (e.g. marriage or divorce). Opportunistic sampling is open to bias, and information on department websites may be outdated. The H-index has limitations and does not discriminate between author order, differentiate between many papers of poor quality and one paper of good quality, or account for self-citations [30]. Because years of active research are determined by an author’s year of first publication, time away for family or maternity leave is not accounted for, a finding that may disproportionately affect women who have their years of active research overestimated. Similarly, institutional or international changes during one’s years of active research are unknown. Regarding leadership positions, no distinctions were made to the hierarchy within leadership positions or time dedicated toward research versus leadership responsibilities. At some institutions, leadership positions are held on a rotation or fixed-term basis. Data for progression through academic rank from the time of first clinical appointment is not known. Sex breakdown with percent of time devoted to research versus community practice, full-time versus part-time employment, time spent in teaching versus time spent in research, and contract versus tenure positions is not known. If more women are in community practice, this would result in a smaller academic footprint.

## Conclusions

Using a cross-sectional analysis, sex disparities among female cardiologists in Canadian academic practice have been found to be prevalent for rank, leadership, and productivity. Women were found to hold fewer leadership roles, have fewer publications, and have a lower academic impact (as judged by the H-index) than male cardiologists at a similar rank. Female assistant professors and associate professors have a lower H-index, demonstrating less research productivity in these ranks. There was an exception to males outperforming females academically at the full professor rank, where women were shown to have higher research productivity (p = 0.002). This exception is potentially indicative of affirmative action policies implemented in recent years. Women may face an academic disadvantage without proper accommodations for family responsibilities or may not have parallel career objectives. As both the true trajectory and desired trajectory of an individual’s academic career path are difficult to characterize, differences in advancement through promotion and publication are likely multifactorial, and consideration should be given to various possibilities. Programs should address disparities within their respective departments, with a long-term goal of enhancing gender equality.

## Appendices

**Table 3:** List of cardiology division and faculty listing websites consulted

University of Saskatchewan
https://medicine.usask.ca/profiles/department-of-medicine/
University of Manitoba
https://umanitoba.ca/medicine/units/cardiac_sciences/cardfellow.html
Memorial University Newfoundland
https://www.med.mun.ca/Discipline-of-Medicine/Regional-Affiliated-Faculty.aspx
https://www.med.mun.ca/Discipline-of-Medicine/st-john-s-affiliated-faculty.aspx
https://www.med.mun.ca/DisciplineofMedicine/medicine-faculty.aspx
Dalhousie University
https://medicine.dal.ca/departments/department-sites/medicine/divisions/cardiology/our-people.html
https://medicine.dal.ca/departments/department-sites/medicine/our-people/faculty.html
University of British Columbia
http://www.ubccardio.com/faculty/
https://pediatrics.med.ubc.ca/divisions-centres/cardiology/
University of Alberta
https://www.ualberta.ca/department-of-medicine/divisions/cardiology/faculty.html
https://www.ualberta.ca/pediatrics/divisions/cardiology.html
https://www.ualberta.ca/medicine/about/people/index.html
University of Calgary
https://libin.ucalgary.ca/departments/cardiac-sciences/members
https://libin.ucalgary.ca/about/directory-members
https://www.ucalgary.ca/research/cumming-school-medicine/cardiac-sciences
McMaster University
https://healthsci.mcmaster.ca/medicine/division/cardiology/faculty-and-staff
https://directories.mcmaster.ca/faculty-staff/
https://fhs.mcmaster.ca/pediatrics/cardiology.html
University of Toronto
https://facdir.deptmedicine.utoronto.ca/Default6.aspx?view=1
https://www.paeds.utoronto.ca/faculty/divlist.htm#PaediatricMedicine
http://www.stmichaelshospital.com/programs/heartvascular/about/cardiologist-bios.php
University of Ottawa
https://www.ottawaheart.ca/clinical-department/cardiology
https://www.ottawaheart.ca/clinical-department/cardiac-imaging
https://www.ottawaheart.ca/patients-visitors/find-specialist
https://med.uottawa.ca/pediatrics/about/faculty?cat_2=141
Université de Sherbrooke
https://www.usherbrooke.ca/dep-medecine/personnel/professeurs-denseignement-clinique-par-specialite/#c158856
https://www.usherbrooke.ca/dep-medecine/recherche/professeurs-ayant-des-activites-de-recherche/cardiologie/
Western University
https://www.schulich.uwo.ca/cardiology/people/faculty.html
https://www.schulich.uwo.ca/paediatrics/divisions/cardiology.html
Queens University
https://deptmed.queensu.ca/divisions/cardiology
Université Laval
https://iucpq.qc.ca/en/research/research-themes/cardiology
https://oraweb.ulaval.ca/pls/vrr/gexp_dap.html
https://www.fmed.ulaval.ca/faculte-et-reseau/a-propos-de-la-faculte/departements/departement-de-medecine/
http://www.vrrc.ulaval.ca/fileadmin/ulaval_ca/Images/recherche/bd/chercheur/fac/dept/03020.html
McGill University
https://www.mcgill.ca/cardiology/people
https://www.mcgill.ca/cardiology/staff
Université de Montréal
https://deptmed.umontreal.ca/departement/cardiovasculaire/
https://www.icm-mhi.org/en/health-care-and-services/our-specialists
https://pediatrie.umontreal.ca/enseignants/
https://recherche.umontreal.ca/english/our-researchers/professors-directory/?tx_udemvitrine%5Bsearch_mode%5D=criteria&tx_udemvitrine%5Bc%5D%5B6%5D%5Bo%5D=AND&tx_udemvitrine%5Bc%5D%5B6%5D%5Bf%5D=Discipline_fac&tx_udemvitrine%5Bc%5D%5B6%5D%5Bq%5D=Cardiology
https://www.icm-mhi.org/en/health-care-and-services/clinics-and-services/department-medicine
Northern Ontario School of Medicine
https://www.nosm.ca/faculty/clinical-sciences/faculty-by-rank/
